# Assessment of acid catalytic properties of ferrosilicate MFI zeolite by methanol-to-hydrocarbon conversion†

**DOI:** 10.1039/d4ra05039h

**Published:** 2024-09-12

**Authors:** Hanyoung Park, Gwang-Jin Na, Jeong-Chul Kim, Ryong Ryoo

**Affiliations:** a Department of Chemistry, Korea Advanced Institute of Science and Technology (KAIST) Daejeon 34141 Republic of Korea; b Center for Nanomaterials and Chemical Reactions, Institute for Basic Science (IBS) Daejeon 34141 Republic of Korea; c Department of Energy Engineering, Korea Institute of Energy Technology (KENTECH) Naju Jeollanam-do 58330 Republic of Korea rryoo@kentech.ac.kr; d Department Chemical and Biological Engineering, Hanbat National University 125 Dongseo-daero, Yuseong-gu Daejeon 34158 Republic of Korea jeongchul@hanbat.ac.kr

## Abstract

Four representative synthetic methods were employed to prepare Fe-containing siliceous MFI zeolites. The obtained Fe-MFI zeolites exhibited markedly different catalytic performances in the methanol-to-hydrocarbon (MTH) conversion reaction depending on the type of Fe incorporation within the siliceous framework. The catalytically active Brønsted acid sites were analyzed using pyridine adsorption experiments combined with Fourier transform infrared spectroscopy, providing characteristic signal intensities according to the acid–base interactions. Based on the MTH conversion results and acidity analyses, a suitable synthetic method was identified for the incorporation of Fe within the MFI zeolite framework. However, compared to other catalytic reactions, structural analyses by transmission electron microscopy, ultraviolet-visible spectroscopy, and X-ray absorption spectroscopy were much less conclusive.

## Abstract 2

Four representative synthetic methods were employed to prepare Fe-containing siliceous MFI zeolites. The catalytic properties of these zeolites were evaluated by methanol-to-hydrocarbon reaction.

## Graphical abstract

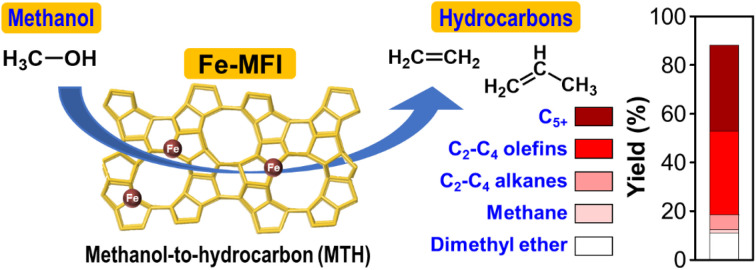

## Introduction

Owing to their unique porous properties, crystalline zeolites are widely used as heterogeneous catalysts in various petrochemical processes.^1,2^ These catalysts exist as silicate frameworks possessing microporous networks and three-dimensional periodicities.^3^ In the zeolitic framework, each Si atom is tetrahedrally coordinated to four oxygen atoms, resulting in a siliceous framework that is electrostatically neutral.^4^ However, in the majority of zeolites, some Si atoms are substituted by Al, resulting in the formation of negatively charged tetrahedral Al(iii) sites.^5^ These negative charges are typically compensated for by the adsorption of Na^+^ or ammonium cations, which can be exchanged for protons to create Brønsted or Lewis acidic sites. Notably, these acidic sites can promote various chemical reactions, including isomerization, alkylation, and hydrocarbon cracking reactions.^6–9^

The incorporation of other transition metals into siliceous frameworks has also been examined, with example metals including Ti, Sn, V, and Fe.^10–13^ Such metal-incorporated zeolites, which are known as metallosilicates or heteroatom zeolites, have been identified as important selective- and partial-oxidation catalysts due to the coordination bonding and redox properties of the transition metals.^14,15^ When a heteroatom in the +3 oxidation state is incorporated, the resulting metallosilicate framework exhibits acidic properties, rendering these zeolites potential catalysts for organic reactions that require a mild Brønsted acidity.^16,17^

Among the various heteroatom zeolites reported to date, the ferrosilicate MFI zeolite (ZSM-5), which is commonly referred to as Fe-MFI, has attracted particular attention in the field of zeolite catalysis due to its moderately strong Brønsted acid sites and its potential to promote the Fe(ii)/Fe(iii) redox transition.^17,18^ However, the incorporation of Fe species is complicated by the low chemical affinity between the Fe metal atoms and the silicate frameworks.^19,20^ Consequently, the low-affinity Fe tends to form extra-framework Fe oxide species during zeolite synthesis. To overcome this problem, extensive studies have been performed into Fe-MFI zeolites, focusing on the thorough mixing of Fe precursor solutions with pre-dissolved silica sources under acidic aqueous conditions prior to silicate gelation with structure-directing agents (SDAs; *e.g.*, tetrapropylammonium hydroxide (TPAOH) or tetrapropylammonium bromide (TPABr) plus NaOH).^21–24^ This approach was aimed at preventing the formation of ferric hydroxide domains, which can be converted into extra-framework iron oxide particles. However, Fe-MFI zeolites have also been prepared by the addition of an Fe source after silicate gelation.^25^ This ongoing debate into the most appropriate synthetic pathway therefore highlights the importance of the thermodynamically metastable nature of zeolites, wherein the structural formation is influenced by a delicate balance between thermodynamic and kinetic factors.^26^ Notably, the state of Fe in the synthesized zeolite (*i.e.*, extra-framework or Fe within the framework) can be influenced by a range of factors, including the reactivity of the Fe precursor, the timing of its addition, the pH of the mixture, and the polymeric state of the silica source.

The identification of Fe species within zeolites requires the use of various characterization techniques, including ultraviolet-visible (UV-vis) spectroscopy, Mössbauer spectroscopy, and X-ray absorption spectroscopy (XAS). Previous literature has suggested that the Fe species present in the resulting zeolites exist predominantly as isolated framework Fe(i), extra-framework Fe(ii), mononuclear Fe(iii), and ion-exchanged Fe(iv) species. However, establishing a clear relationship between the state of the Fe species and the catalytic properties of the synthesized Fe-MFI zeolites remains a challenge.

Thus, in the current study, four representative synthetic methods currently available for Fe-MFI synthesis are selected to prepare a selection of zeolites. Subsequently, the catalytic properties of the synthesized Fe-MFI samples are evaluated in the methanol-to-hydrocarbon (MTH) conversion reaction to assess their catalytic efficiencies. In addition, the Brønsted acidities of the zeolites are quantitatively analyzed using Fourier-transform infrared (FT-IR) spectroscopy with pyridine as a probe molecule. Ultimately, the aim of this study is to identify the variations in acidity resulting from the different synthetic methods, and to establish correlations with the MTH activity. Moreover, following the catalytic MTH reaction and FT-IR analysis, the zeolite structures are thoroughly characterized using various physicochemical techniques, including X-ray diffraction (XRD), Ar adsorption isotherms, ^29^Si magic-angle spinning solid-state nuclear magnetic resonance (^29^Si MAS NMR) spectroscopy, UV-vis spectroscopy, X-ray absorption near edge structure (XANES) analysis, scanning electron microscopy (SEM), scanning transmission electron microscopy (STEM), and elemental mapping combined with energy dispersive spectroscopy (EDS). The obtained results are comprehensively integrated to evaluate the performances of the prepared Fe-MFI catalysts.

## Experimental

### Synthetic routes for Fe-MFI

#### Method (1) addition of Fe nitrate into acidic sol obtained from HNO_3_ + TEOS

Tetraethyl orthosilicate (TEOS, 4.00 g, Junsei) was mixed with Fe(NO_3_)_3_·9H_2_O (0.155 g, Sigma-Aldrich) in deionized water (9.0 g) with HNO_3_ solution (0.014 g, Daejung, 61%) until hydrolysis was complete (∼1 h), and a homogeneous solution was obtained. After stirring for 1 h at 300 rpm in a polypropylene (PP) bottle, a TPAOH solution (5.73 g, 22.5 wt% in H_2_O, TCI) was added. The mixture was vigorously shaken by hand for several minutes to give a mixture with the following composition: 100 SiO_2_/33TPAOH/2Fe(NO_3_)_3_·9H_2_O/4000H_2_O/0.73HNO_3_. After aging for 12 h at 60 °C under stirring (300 rpm), the obtained mixture was transferred to a Teflon-lined autoclave, which was heated for 12 h at 170 °C until the zeolite precipitated. The zeolite product was collected by centrifugation, washed with deionized water, dried in oven at 100 °C, and finally calcined at 580 °C under a flow of air. Subsequently, the calcined zeolite was ion-exchanged three times with a 0.1 M aqueous solution of NH_4_NO_3_ at 25 °C, and calcined again at 580 °C for 4 h under air. Elemental analysis was performed by inductively coupled plasma atomic emission spectroscopy (ICP-AES, PerkinElmer OPTIMA 4300 DV). This Fe-MFI sample was denoted as MFI_TPAOH/(TEOS+Fe)_, which represents the mixing order of the components during this procedure.

#### Method (2) Fe nitrate into basic gel from TPAOH + TEOS

TEOS (4.00 g) was mixed thoroughly with the TPAOH solution (22.5 wt% in H_2_O, 5.73 g) in a PP bottle for 10 min to obtain the silicate gel. The gel was then added to an aqueous solution (9.0 g) containing Fe(NO_3_)_3_·9H_2_O (0.155 g), and the mixture was stirred (300 rpm) for 1 h at 25 °C. The molar ratio composition of this mixture was 100SiO_2_/33TPAOH/2Fe(NO_3_)_3_·9H_2_O/4000H_2_O. After aging this mixture for 12 h at 60 °C, the subsequent steps were as described above for the MFI_TPAOH/(TEOS+Fe)_ specimen (method (1)). The obtained zeolite was denoted as MFI_Fe/(TEOS+TPAOH)_.

#### Method (3) Fe nitrate into sodium silicate + TPABr

Fumed silica (0.500 g, Sigma-Aldrich) was mixed with a solution of NaOH (0.280 g, Daejung) in deionized water (6.0 g), and aged at 60 °C for 3 h to obtain a highly pure sodium silicate solution (*i.e.*, with a low Fe content). Subsequently, an aqueous solution (2.0 g) containing TPABr (0.233 g, 99.4%, TCI) was added to the sodium silicate solution, followed by the dropwise addition of an aqueous solution (4.0 g) containing Fe(NO_3_)_3_·9H_2_O (67.2 mg) under stirring (300 rpm). A 10 wt% sulfuric acid solution (2.23 g) was then added dropwise to adjust the acidity of the solution. The molar ratio composition of this mixture was 95SiO_2_/40Na_2_O/10TPABr/26H_2_SO_4_/1.9Fe/9000H_2_O. This mixture was aged for 12 h at 60 °C, and the remainder of the synthetic procedure followed method (1), with the exception that the autoclaving step was carried out for 2 d at 150 °C. The resulting sample was denoted as MFI_Fe/(NaSil+TPABr)_.

#### Method (4) addition of TPABr after Fe nitrate + sodium silicate

A sodium silicate was prepared as described in method (3), and added immediately to a solution of Fe(NO_3_)_3_·9H_2_O (67.2 mg) in deionized water (4.0 g). The resulting mixture was immediately shaken vigorously by hand for several minutes, then stirred magnetically at 300 rpm for 2 h. After the initial 1 h of stirring, the mixture in the PP bottle was added to an aqueous TPABr solution (0.233 g TPABr in 2.0 g deionized water). Once the stirring time was complete, 10 wt% sulfuric acid (2.23 g) was added dropwise with stirring (300 rpm) to adjust the pH to 12. The molar composition of the resulting mixture and the remainder of the synthetic procedure were as described for method (3). The synthesized sample was denoted as MFI_TPABr/(NaSil+Fe)_.

### MTH reaction measurements

The MTH reactions were performed in a stainless-steel fixed-bed reactor using Fe-MFI (50 mg) as the catalyst. All catalyst samples were initially activated in a N_2_ flow at 500 °C for 3 h in the reactor. The reactor temperature was then lowered to 400 °C, and the gas was switched to a mixture of N_2_ and methanol vapor. The methanol feeding rate was controlled to a weight hourly space velocity (WHSV) of 10 g_MeOH_ g_MFI_^−1^ h^−1^, by bubbling N_2_ gas (20 mL min^−1^) through liquid methanol at ∼31 °C. The reaction products were analyzed using an online gas chromatograph (GC, YL6500) equipped with a flame ionization detector and a GC column (HP-plot *Q*, *l* = 50 m, i.d. = 0.32 mm, *t* = 1 μm, Agilent J&W). After the reaction, the catalyst samples were analyzed using thermogravimetry (Q50, TA Instruments).

The ability of the catalyst to regenerate was investigated after the MTH measurements under a high WHSV (50 h^−1^) to ensure for rapid deactivation. After each MTH run, the catalyst sample was regenerated by calcination under a dilute flow of O_2_ (2 vol% O_2_ in N_2_). The catalyst was then heated to 500 °C over a period of 2 h, and subjected to calcination at this temperature for a further 1 h.

### Brønsted acid analysis by FT-IR

The Brønsted acid site content was calculated using FT-IR (JASCO FTIR-6100) with pyridine as the probe molecule.^27–29^ A total of 40 scans were employed at a resolution of 4 cm^−1^. Prior to carrying out the FT-IR measurements, each Fe-MFI sample was pressed into a self-supported wafer, and the obtained pellets were placed in a lab-made IR cell and degassed at 400 °C for 4 h. After cooling to 150 °C, pyridine was allowed to adsorb on the degassed sample for 30 min. Subsequently, the zeolite samples were evacuated at 150, 300, or 500 °C for 1 h to observe the effect of temperature on the desorption of pyridine. To quantify the Brønsted acid sites, the peak areas were compared after normalization of the spectra. The commercial aluminosilicate ZSM-5 (CBV8014, Zeolyst, Si/Al = 40) was employed as a reference material for quantification after calcination under air at 580 °C. Thus the molar extinction coefficient of the Brønsted acid sites was calculated as *εB* = 1.38 cm mmol^−1^.^30^

### Structural characterization of the zeolites

The XRD patterns of the zeolite specimens were measured in the 2*θ* range of 5–35°, with a step size of 0.02° and a scan speed of 4° min^−1^. For this purpose, a Rigaku Smartlab diffractometer was employed with Cu Kα radiation (40 kV, 30 mA). The relative crystallinity of Fe-MFIs were obtained by calculating the ratio between the integrated XRD peak areas of synthesized zeolites and that of purely siliceous zeolite.^31–33^ The specific surface areas of the samples were determined according to the Brunauer–Emmett–Teller (BET) method, wherein the calculations were based on the adsorption data recorded in the range of 0.05 < *P*/*P*_0_ < 0.2 for argon adsorption isotherms measured at −186 °C (Quantachrome Autosorb iQ). Prior to performing the adsorption measurements, all samples were degassed at 300 °C for 3 h. The ^29^Si MAS NMR spectra were obtained at a spin rate of 12 kHz using a Bruker Avance 400WB spectrometer with tetramethylsilane as the internal standard. The spectra were measured using a π/2 pulse width of 4 μs with a relaxation delay time of 5 s, and a total of 10 000 scans. The SEM images were recorded using an FEI Verios 460 L microscope at an acceleration voltage of 1 kV. The STEM images and EDS data were obtained at an acceleration of 300 kV using an FEI Titan ETEM G2 at Institute for Basic Science (IBS) and FEI Titan cubed G2 60-300 at the KAIST Analysis Center for Research Advancement (KARA). The UV-vis spectra were recorded using a PerkinElmer Lambda 1050 spectrophotometer at KARA, while the XANES analyses of the pre-dehydrated samples (evacuated at 300 °C) were performed in transmission mode at the Fe K-edge (Pohang Accelerator Laboratory).

## Results and discussion

### Influence of the synthetic method on the catalyst structure and activity

Four types of Fe-MFI samples with bulk crystalline morphologies were synthesized using different synthetic procedures. In all synthetic approaches examined herein, the starting gels were prepared using a Si/Fe molar ratio of 50. According to the ICP-AES results presented in Table 1, the actual Si/Fe ratios in the synthesized zeolites were consistent with the amount of incorporated Fe, within a variation range of 50 ± 6. Furthermore, ICP-AES analysis also confirmed that treatment with NH_4_NO_3_ successfully removed sodium from the samples, which is necessary to avoid blockage of the acidic sites generated by the Fe atoms within the framework. The sample notations employed in Table 1 represent the individual synthetic methods, and in particular, they reflect the mixing orders of the synthetic components.

**Table 1:** Fe-MFI zeolites synthesized by different methods

Notation by mixing order^a^	Synthesis section	Silica source	Si/Fe by ICP-AES
MFI_TPAOH/(TEOS+Fe)_	(1)	TEOS	52
MF_Fe/(TEOS+TPAOH)_	(2)	TEOS	44
MFI_Fe/(NaSil+TPABr)_	(3)	Water glass	46
MFI_TPABr/(NaSil+Fe)_	(4)	Water glass	55

a A/(B + C) in subscript indicates that A was added in the synthesis composition after B and C were mixed.

**Fig. 1:**
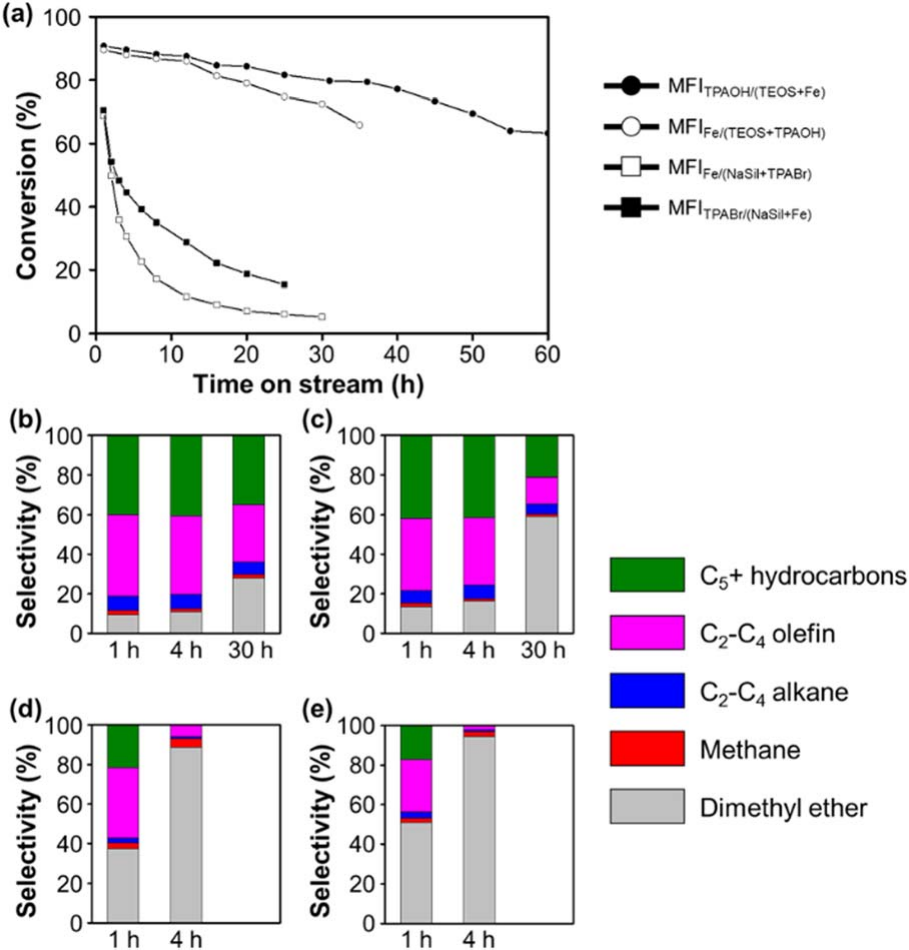
The MTH reaction catalytic performance of the Fe-MFI zeolites. (a) Conversion rates of methanol as a function of reaction time and (b–e) selectivity of each hydrocarbon products by (b) MFI_TPAOH/(TEOS+Fe)_, (c) MFI_Fe/(TEOS+TPAOH)_, (d) MFI_Fe/(NaSil+TPABr)_ and (e) MFI_TPABr/(NaSil+Fe)_. Reaction conditions were as follows: 50 mg catalyst, 400 °C, and WHSV of methanol = 10 h^−1^.

Fig. 1 shows the MTH conversion yields and product selectivities achieved using the four prepared Fe-MFI zeolites. It can be seen that the MFI_TPAOH/(TEOS+Fe)_ zeolite exhibits a high conversion and selectivity for C_2+_ hydrocarbons until reaching a time-on-stream (TOS) of 55 h. Notably, the observed high catalytic yield and slow catalyst deactivation were comparable to those described previously for aluminosilicate MFI zeolites.^34^ After the MFI_TPAOH/(TEOS+Fe)_ zeolite, MFI_Fe/(TEOS+TPAOH)_ zeolite was the second best catalyst, exhibiting a comparable C_2+_ yield over 30 h TOS. In contrast, the MFI_Fe/(NaSil+TPABr)_ and MFI_TPABr/(NaSil+Fe)_ catalysts, which were prepared using sodium silicate, exhibited extremely low C_2+_ yields, along with significant degrees of catalyst deactivation. Thus, the catalytic efficiencies for the MTH conversion reaction appeared to decrease in the order of: MFI_TPAOH/(TEOS+Fe)_ ≥ MFI_Fe/(TEOS+TPAOH)_ >> MFI_Fe/(NaSil+TPABr)_ ≈ MFI_TPABr/(NaSil+Fe)_.

According to the thermogravimetric analysis (Fig. S1†), the MFI_TPAOH/(TEOS+Fe)_ catalyst collected after 55 h of TOS contained a combustible coke content amounting to 8.2% of the initial zeolite weight. The color of the catalyst also changed from white to dark greenish-gray during the TOS, although these changes were both reversible upon flowing a mixture of 2% O_2_ in N_2_ through the system at 500 °C. Moreover, the C_2+_-producing ability and catalytic lifetime were regenerated to ∼80% following such treatment; this result is comparable to a previous report, describing 90% regeneration of an aluminosilicate MFI catalyst using 2% O_2_ at 550 °C after MTH operation.^35^ However, upon heating the spent MFI_TPAOH/(TEOS+Fe)_ catalyst under air at 550 °C in an attempt to maximize the regeneration capacity, the catalytic activity decreased, indicating possible Fe demetallation from the frameworks at high temperatures (Fig. S2†).^36^

### Effect of Fe incorporation

Ferric nitrate is one of the most common Fe sources in the synthesis of Fe-MFIs. However, as shown in Fig. 1, the addition of an Fe source caused a dramatic difference in the catalytic properties of the MTH. As previously reported, the addition of conventional salt-type Fe precursors (*e.g.*, chloride and nitrate) to the gel under high pH conditions can cause the rapid precipitation of poorly soluble Fe hydroxide particles.^22,23^ Once precipitated, the Fe hydroxide particles can be encapsulated inside or agglomerated outsize the zeolite particles upon their hydrothermal crystallization. Subsequently, the Fe hydroxide is transformed into iron oxide particles during high-temperature zeolite calcination under air or O_2_. Under such circumstances, it is anticipated that it will be difficult to achieve single-atomic Fe incorporation to form Brønsted acid sites, thereby accounting for the fact that the MFI_Fe/(NaSil+TPABr)_ and MFI_TPABr/(NaSil+Fe)_ zeolites exhibited poor catalytic performances in the MTH conversion to generate C_2+_ products (Fig. 1d and e).

As outlined in Table 1, the MFI_Fe/(TEOS+TPAOH)_ zeolite was synthesized by adding Fe nitrate to a mixture of TEOS, TPAOH, and H_2_O at pH 12. This pH was lower than that of the Na silicate solution used to prepare the MFI_Fe/(NaSil+TPABr)_ and MFI_TPABr/(NaSil+Fe)_ systems (*i.e.*, pH 14), but was still sufficiently high to promote the rapid precipitation of iron hydroxides. It was expected that the formation of extra-framework Fe species under these conditions would result in a negligible conversion to C_2+_ products. However, in contrast, a relatively high C_2+_ yield was obtained (Fig. 1), thereby indicating that the effect of a high pH cannot fully explain the MTH catalytic performance of MFI_Fe/(TEOS+TPAOH)_. Thus, to account for the observed results, it was considered that the choice of silica source (*e.g.*, TEOS with monomeric Si atoms) could influence the atomic incorporation of Fe.^13^ In particular, in the case of the MFI_TPAOH/(TEOS+Fe)_ sample, the aqueous Fe(NO_3_)_3_ solution was sufficiently acidic to promote the hydrolysis of TEOS into a more reactive monomeric ≡Si–OH species. This reactive silica species can then immediately combine with a hydrated Fe(iii) cation, resulting in the atomically disperse incorporation of Fe atoms within the polymerizing silicate network. This speculation may be extended to the case of the MFI_Fe/(TEOS+TPAOH)_ system, with the exception that Fe incorporation occurs less effectively because of the more rapid polymerization of the silica source in the presence of TPAOH. In contrast to the cases using monomeric TEOS, preparation of the MFI_Fe/(NaSil+TPABr)_ and MFI_TPABr/(NaSil+Fe)_ zeolites employed polymeric sodium silicates. Ultimately, this results in the less effective incorporation of Fe due to the difficulties associated with inserting Fe into the Si–O–Si network.

During preparation of the catalysts, sufficient mixing of the starting materials is also critical for ensuring the optimal catalytic performance. For example, in the aforementioned synthesis of MFI_Fe/(TEOS+TPAOH)_, an Fe(NO_3_)_3_ solution was rapidly added to a homogeneous sol composed of H_2_O, TEOS, and TPAOH with vigorous mechanical stirring. It was assumed that this rapid mixing ensured uniform incorporation of the Fe precursor into the silica source. The resulting zeolite catalyst exhibited a good conversion to C_2+_ products over 30 h TOS, as shown in Fig. 1a. To confirm the effect of such vigorous mixing, another zeolite sample was prepared using identical starting materials and compositions, but with the dropwise addition of the Fe(NO_3_)_3_ solution to the H_2_O/TEOS/TPAOH mixture under magnetic stirring. In this case, a sudden increase in the mixture viscosity was observed during addition of the Fe precursor, leading to apparent gelation. This indicated that rapid polymerization of the silicates occurred, which could result in an undesirable heterogeneous distribution of the Fe precursor. Indeed, the obtained zeolite exhibited an extremely low catalytic performance in the MTH conversion reaction, similar to a previous report from Meng *et al.* (Fig. S3†).^29^ This result demonstrates that the incorporation of Fe into the MFI zeolite is extremely sensitive to the synthetic procedure employed during zeolite preparation.

### Quantification of the Brønsted acid sites by pyridine adsorption and FT-IR experiments

**Fig. 2:**
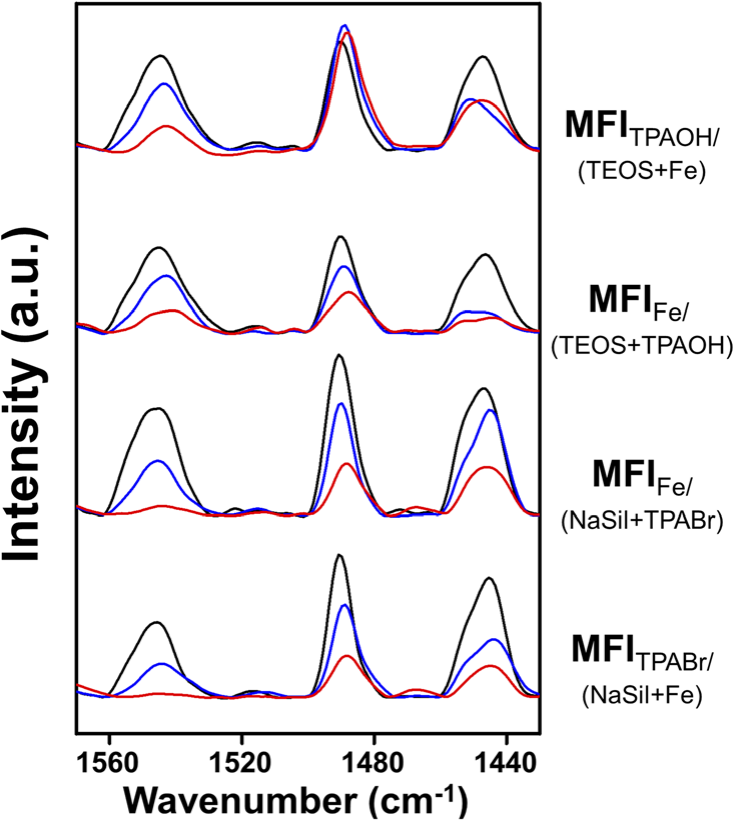
FT-IR spectra of pyridine-adsorbed Fe-MFI samples. Spectra were measured after the desorption of pyridine at 150 °C (black line), 300 °C (blue line) and 500 °C (red line) for 1 h.

Subsequently, the Brønsted acid sites were analyzed using pyridine adsorption combined with FT-IR experiments to determine if the acid concentration correlated with the MTH catalytic performance. As shown in Fig. 2, the peaks observed at 1545 cm^−1^ for the four Fe-MFI zeolites corresponded to the characteristic band observed for Brønsted acid sites.^37^ After pyridine desorption at 150 °C, all zeolites appeared to contain significant and analogous amounts of acid sites. However, after desorption at 300 and 500 °C, differences were observed between the samples, suggesting variations in their acidic strengths.^28,38^

For a more accurate analysis, quantification of the Brønsted acid sites was performed, and the results are summarized in Table 2. Using commercial ZSM-5 as the reference material to determine the molar extinction coefficient (Fig. S4†), the number of Brønsted acid sites in each Fe-MFI zeolite catalyst was calculated. As shown in Table 2, the number of Brønsted acid sites do not correlate with the MTH activity. For example, although MFI_Fe/(NaSil+TPABr)_ contained the highest number of acid sites, its MTH catalytic performance was rapidly deactivated. However, it was found that the amount of pyridine desorbed at high temperatures was related to catalytic performance. More specifically, after pyridine desorption at 300 °C, the MFI_TPAOH/(TEOS+Fe)_ and MFI_Fe/(TEOS+TPAOH)_ catalysts retained greater amounts of adsorbed pyridine than the MFI_Fe/(NaSil+TPABr)_ and MFI_TPABr/(NaSil+Fe)_ catalysts; this phenomenon was more pronounced after desorption at 500 °C. Based on the obtained results, the number of acid sites determined by the high-temperature pyridine desorption experiments decreased in the order of MFI_TPAOH/(TEOS+Fe)_ ≥ MFI_Fe/(TEOS+TPAOH)_ ≫ MFI_Fe/(NaSil+TPABr)_ ≈ MFI_TPABr/(NaSil+Fe)_, consistent with the decreasing order of catalytic performance described above. Notably, a linear correlation was observed between the C_2+_ hydrocarbon yield and the number of Brønsted acid sites, as shown in Fig. S5.† This result implies that the catalyst must possess a certain degree of acidity to promote the MTH reaction, and to retain pyridine at high temperatures. Consequently, MFI_TPAOH/(TEOS+Fe)_ and MFI_Fe/(TEOS+TPAOH)_ were considered to be more catalytically active than MFI_Fe/(NaSil+TPABr)_ and MFI_TPABr/(NaSil+Fe)_.

**Table 2:** Amount of Brønsted acid sites of Fe-MFI zeolites, after the desorption of pyridine at 150 °C, 300 °C and 500 °C

Zeolite samples	Brønsted acid site (μmol g^−1^ of zeolite)		
	150 °C	300 °C	500 °C
MFI_TPAOH/(TEOS+Fe)_	157	103	37
MF_Fe/(TEOS+TPAOH)_	145	92	36
MFI_Fe/(NaSil+TPABr)_	182	81	5
MFI_TPABr/(NaSil+Fe)_	114	53	N/A

### Fe mapping in the zeolite structure

**Fig. 3:**
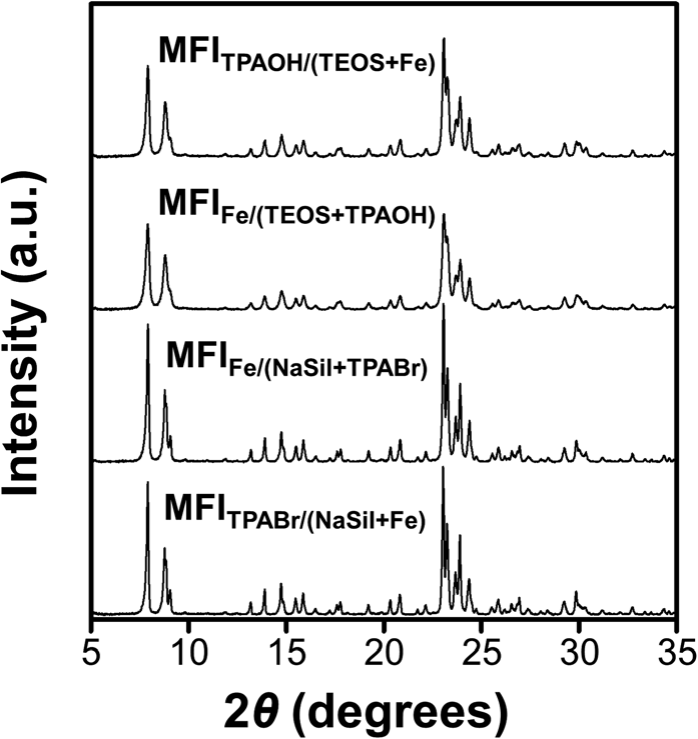
Powder XRD patterns of Fe-MFI zeolite samples.

The XRD patterns shown in Fig. 3 indicate that all Fe-MFI samples investigated herein are fully crystalline MFI zeolites containing no detectable amounts of amorphous silica or other impurities. No XRD peaks corresponding to the iron oxide phases were detected, and the synthesized zeolites exhibited an average crystallinity of 88% compared to the pure siliceous MFI zeolite (Table S1†). The high crystallinities of the synthesized Fe-MFI zeolites were also confirmed by ^29^Si MAS NMR spectroscopy and SEM imaging at low magnifications (Fig. S5 and S6†). Moreover, the samples possessed similar surface areas ranging from 320 to 350 m^2^ g^−1^, which are in good agreement with the typical values for highly crystalline MFI zeolites.^39^

**Fig. 4:**
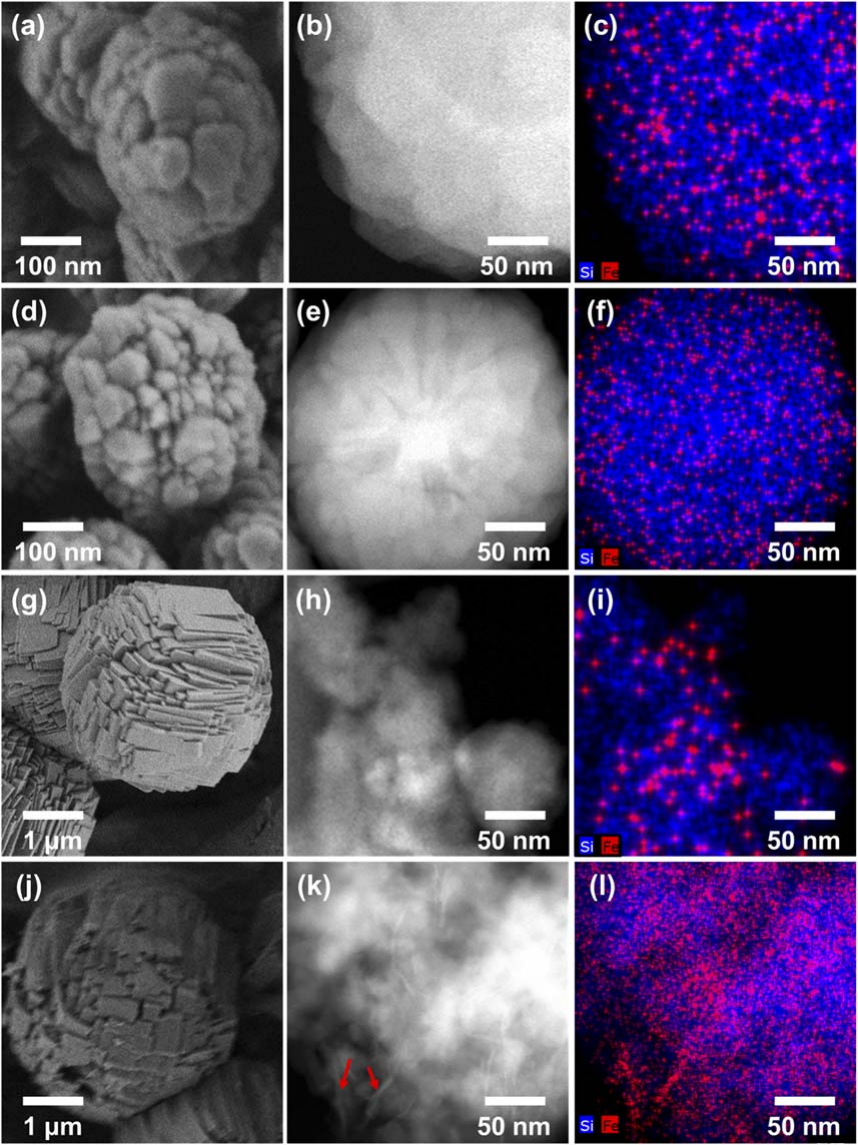
SEM images (left), STEM images (center) and EDS mapping analysis images (right) of (a–c) MFI_TPAOH/(TEOS+Fe)_, (d–f) MFI_Fe/(TEOS+TPAOH)_, (g–i) MFI_Fe/(NaSil+TPABr)_ and (j–l) MFI_TPABr/(NaSil+Fe)_.

Fig. 4 shows the SEM, STEM, and EDS images recorded to investigate the zeolite crystal morphologies and the spatial distributions of Fe in the various structures. It can be seen that the zeolite particles were significantly larger for the sodium silicate-based zeolites (2–3 μm) than for the TEOS-based zeolites (200–400 nm, Fig. S7†). According to previous literature, zeolite crystal growth is believed to be influenced by the presence of Na^+^ ions, which appear to increase the zeolite thickness.^40^ Despite the large differences in the particle diameters recorded for the various Fe-MFI samples, Fe elemental mapping by STEM-EDS showed that all specimens contained highly dispersed Fe domains, mostly contained within the zeolite particles. However, it was difficult to accurately distinguish the sizes of the individual Fe domains/particles owing to the limited resolution resulting from the particles being obscured by the silica background. Nevertheless, it was deduced that the sodium silicate-based Fe-MFIs exhibited larger Fe domains than the TEOS-based zeolites. Furthermore, the STEM image (Fig. 4k) and the Fe mapping results (Fig. 4l) show the presence of rod-like Fe domains (indicated by arrows), thereby demonstrating that the different catalytic activities and acidities of the samples could be influenced by unmeasurable factors, such as the presence of amorphous Fe-silica complexes. These results therefore demonstrate that the TEOS-based systems possess high dispersions of Fe, which can promote superior catalytic activities, and account for the previously described order of catalytic performance.

### Fe coordination environments

**Fig. 5:**
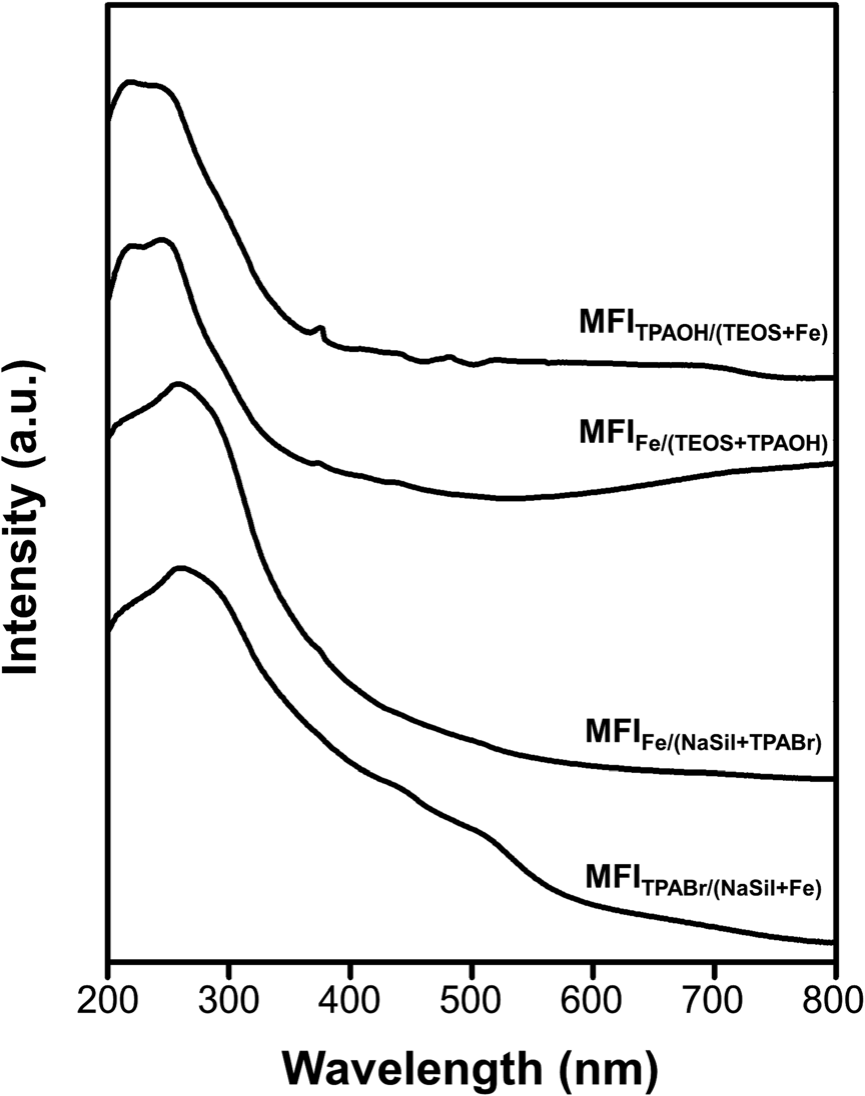
Diffuse-reflectance UV-vis spectra of Fe-MFI zeolite samples.

The above results revealed that the single-atomic incorporation of Fe is critical for the generation of Brønsted acid sites to catalyze the MTH conversion process. However, it remains unclear whether the Fe atoms are tetrahedrally coordinated to neighboring –OSi species within the zeolite framework, or whether they exist as an extra-framework species with an octahedral coordination. Thus, UV-vis spectroscopy was performed to obtain information regarding coordination of the Fe atoms. As presented in Fig. 5, the spectra recorded for the MFI_TPAOH/(TEOS+Fe)_ and MFI_Fe/(TEOS+TPAOH)_ catalysts exhibit intense peaks centered at 210 and 240 nm. These electronic charge transfer peaks originate from the t_1_ → t_2_ and t_1_ → e transitions of tetrahedrally coordinated Fe(iii) atoms.^41,42^ These signals were therefore considered to be characteristic of the single atomic tetrahedral Fe^3+^ bonded to four neighboring –OSi species within the zeolite framework. However, in the cases of the MFI_Fe/(NaSil+TPABr)_ and MFI_TPABr/(NaSil+Fe)_ catalysts, the above peak shifted to 270 nm, shouldered, and tailed to 550 nm. This is characteristic of an octahedral Fe(iii) symmetry of a single-atomic extra-framework species or individual iron oxide particles.^38^ Additionally, the relatively high intensity of the Q_3_ peak in the ^29^Si MAS NMR spectra of Fe-MFI zeolites (Fig. S6†) also supports the presence of framework Fe species. The Q_3_/Q_4_ ratios for the MFI_TPAOH/(TEOS + Fe)_ and MFI_Fe/(TEOS + TPAOH)_ samples were determined to be ∼5–6%, while for the MFI_Fe/(NaSil+TPABr)_ and MFI_TPABr/(NaSil+Fe)_ samples, the number of Q_3_ groups (*i.e.*, silanol groups) was expected to be negligibly small (∼1–2%) owing to the presence of extra-framework Fe moieties. Based on these interpretations, the TEOS-based Fe-MFI zeolites were considered to contain relatively high proportions of single-atomic tetrahedral Fe^3+^ sites incorporated inside the zeolite framework, while the sodium silicate-based Fe-MFI zeolites appeared to possess significant Fe contents outside the zeolite framework.

XANES analysis was also performed to analyze Fe coordination more precisely in the Fe-MFI zeolites (Fig. S7†). A notable feature of each XANES result is a pre-edge peak centered at 7114 eV, which is often attributed to X-ray absorption through the 1s → 3d electronic transition of 3d transition metals with tetrahedral coordination.^28,43^ Consequently, the intensity of this pre-edge peak is often used to characterize coordination symmetries in various 3d transition metals. For example, in the case of Ti-MFI zeolites, the peak corresponding to the tetrahedral framework Ti species is significantly more intense than that of the extra-framework octahedral Ti species. However, compared with these previously described Ti-MFI systems, the XANES pre-edge peaks recorded for the four Fe-MFI zeolites (Fig. S7†) do not indicate such remarkable differences. Indeed, the pre-edge peaks were only slightly more intense for MFI_TPAOH/(TEOS+Fe)_ and MFI_Fe/(TEOS+TPAOH)_ than for MFI_Fe/(NaSil+TPABr)_ and MFI_TPABr/(NaSil+Fe)_. Although the edge region at 7130–7150 eV exhibited some differences between the sodium silicate-based Fe-MFIs and the TEOS-based samples, the XANES results could not be correlated with the MTH catalytic activities, Brønsted acid sites, TEM images, or UV-vis spectra discussed above.

## Conclusions

In this study, a selection of Fe-containing siliceous MFI (Fe-MFI) zeolites were prepared using various synthetic approaches, and their catalytic performances in the methanol-to-hydrocarbon (MTH) conversion reaction were found to depend on the preparation method employed. The obtained results indicated that the status of Fe in the zeolite (*i.e.*, framework or extra-framework) is sensitive to the synthetic conditions, such as the monomeric or polymeric state of the silica source, and the mixing mode between the silica source and the Fe precursor. Considering the role of the Brønsted acid sites in defining the catalytic activity, the optimal performance was obtained for the tetraethyl orthosilicate (TEOS)-based MFI_TPAOH/(TEOS+Fe)_ system. More specifically, using this zeolite as a catalyst, the yield of C_2+_ hydrocarbon products obtained from the MTH conversion was comparable to that achieved using an aluminosilicate MFI zeolite, with the exception that the spent Fe-MFI catalyst had to be regenerated carefully to prevent Fe demetallation from the framework. Notably, the synthetic procedure employed to obtain the MFI_TPAOH/(TEOS+Fe)_ zeolite features homogeneous incorporation of the Fe precursor into a solution of monomeric ≡Si–OH species prior to polymerization of the silica source. Based on the resulting MTH performance and a detailed structural characterization, it was concluded that this zeolite possessed the highest degree of framework Fe incorporation among the Fe-MFI zeolites investigated herein.

## Data availability

The data supporting this paper are included in the main article and the ESI.†

## Author contributions

Conceptualization: R. R., investigation: R. R. and H. P., methodology: H. P., G.-J. N. and J.-C. K., writing: R. R. and H. P., review and editing: J.-C. K. All authors have read and agreed to the published version of the manuscript.

## Conflicts of interest

There are no conflicts to declare.

## Supplementary Material

RA-014-D4RA05039H-s001

## References

[cit1] Cundy C. S., Cox P. A. (2003). Chem. Rev..

[cit2] Cundy C. S., Cox P. A. (2005). Microporous Mesoporous Mater..

[cit3] Van Donk S., Janssen A. H., Bitter J. H., De Jong K. P. (2003). Catal. Rev.: Sci. Eng..

[cit4] Liu G., Liu J., He N., Miao C., Wang J., Xin Q., Guo H. (2018). RSC Adv..

[cit5] Corma A. (1995). Chem. Rev..

[cit6] Kim J., Han S. W., Kim J. C., Ryoo R. (2018). ACS Catal..

[cit7] Christensen C. H., Johannsen K., Schmidt I., Christensen C. H. (2003). J. Am. Chem. Soc..

[cit8] Kim J. C., Cho K., Ryoo R. (2014). Appl. Catal., A.

[cit9] Rahimi N., Karimzadeh R. (2011). Appl. Catal., A.

[cit10] Notari B. (1991). Stud. Surf. Sci. Catal..

[cit11] Hammond C. (2017). Stud. Surf. Sci. Catal..

[cit12] Sen T., Rajamohanan P. R., Ganapathy S., Sivasanker S. (1996). J. Catal..

[cit13] Ratnasamy P., Kumar R. (1991). Catal. Today.

[cit14] Michalkiewicz B. (2004). Appl. Catal., A.

[cit15] Koekkoek A. J. J., Kim W., Degirmenci V., Xin H., Ryoo R., Hensen E. J. M. (2013). J. Catal..

[cit16] Yu Q., Feng Y., Tang X., Yi H., Zhao S., Gao F., Zhou Y., Zhang Y., Zhuang R. (2020). Microporous Mesoporous Mater..

[cit17] Zhang J., Tang X., Yi H., Yu Q., Zhang Y., Wei J., Yuan Y. (2022). Appl. Catal., A.

[cit18] Miyake K., Hirota Y., Ono K., Uchida Y., Miyamoto M., Nishiyama N. (2017). New J. Chem..

[cit19] Yang G., Pidko E. A., Hensen E. J. M. (2013). J. Phys. Chem. C.

[cit20] Luo H. Y., Lewis J. D., Román-Leshkov Y. (2016). Annu. Rev. Chem. Biomol. Eng..

[cit21] Yuranov I., Bulushev D. A., Renken A., Kiwi-Minsker L. (2007). Appl. Catal., A.

[cit22] Szostak R., Nair V., Thomas T. L. (1987). J. Chem. Soc., Faraday Trans..

[cit23] Gu J., Jin Y., Zhou Y., Zhang M., Wu Y., Wang J. (2013). J. Mater. Chem. A.

[cit24] Marosi L. , Stabenow J. and Schwarzmann M., DE Pat., 2831631, 1980

[cit25] Zhang Q., Guo Q., Wang X., Shishido T., Wang Y. (2006). J. Catal..

[cit26] Bae J., Dusselier M. (2022). Chem. Commun..

[cit27] Selli E., Rossetti I., Meloni D., Sini F., Forni L. (2004). Appl. Catal., A.

[cit28] Lee K. Y., Lee S. W., Ihm S. K. (2014). Ind. Eng. Chem. Res..

[cit29] Meng L., Zhu X., Mezari B., Pestman R., Wannapakdee W., Hensen E. J. M. (2017). ChemCatChem.

[cit30] Milina M., Mitchell S., Michels N. L., Kenvin J., Pérez-Ramírez J. (2013). J. Catal..

[cit31] Li Y., Liu D., Liu S., Wang W., Xie S., Zhu X., Xu L. (2008). J. Nat. Gas Chem..

[cit32] Ji Y., Shi B., Yang H., Yan W. (2017). Appl. Catal., A.

[cit33] Su S., Ma H., Chuan X. (2016). Adv. Powder Technol..

[cit34] Kim J., Choi M., Ryoo R. (2010). J. Catal..

[cit35] Barbera K., Sorensen S., Bordiga S., Skibsted J., Fordsmand H., Beato P., Janssens T. V. W. (2012). Catal. Sci. Technol..

[cit36] Campbell S. M., Bibby D. M., Coddington J. M., Howe R. F. (1996). J. Catal..

[cit37] Kim K., Ryoo R., Jang H. D., Choi M. (2012). J. Catal..

[cit38] Meng L., Zhu X., Hensen E. J. M. (2017). ACS Catal..

[cit39] Jo C., Cho K., Kim J., Ryoo R. (2014). Chem. Commun..

[cit40] Kim Y., Kim K., Ryoo R. (2017). Chem. Mater..

[cit41] Bordiga S., Buzzoni R., Geobaldo F., Lamberti C., Giamello E., Zecchina A., Leofanti G., Petrini G., Tozzola G., Vlaic G. (1996). J. Catal..

[cit42] Ribera A., Arends I. W. C. E., De Vries S., Pérez-Ramírez J., Sheldon R. A. (2000). J. Catal..

[cit43] Berlier G., Spoto G., Fisicaro P., Bordiga S., Zecchina A., Giamello E., Lamberti C. (2002). Microchem. J..

