# Supplementary material for: Assessment of acid catalytic properties of ferrosilicate MFI zeolite by methanol-to-hydrocarbon conversion
Source: RSC Adv. 2024 Sep 12;14(40):29006–13. doi: 10.1039/d4ra05039h (PMC11391894; doi:10.1039/d4ra05039h)
Supplement: RA-014-D4RA05039H-s001 [file RA-014-D4RA05039H-s001.pdf]

Supplementary Information (SI) for RSC Advances.
This journal is © The Royal Society of Chemistry 2024

Supporting Information

Assessment of Acid Catalytic Properties of Ferrosilicate MFI Zeolite by Methanol-to-Hydrocarbon Conversion

Hanyoung Park,[†,a,b] *Gwang-Jin Na,*[†,c] *Jeong-Chul Kim,* [d]* *and Ryong Ryoo*[c]*

[a] Department of Chemistry, Korea Advanced Institute of Science and Technology (KAIST), Daejeon 34141, Republic of Korea

[b] Center for Nanomaterials and Chemical Reactions, Institute for Basic Science (IBS), Daejeon 34141, Republic of Korea

[c] Department of Energy Engineering, Korea Institute of Energy Technology (KENTECH), Naju, Jeonnam 58330, Korea

[d] Department Chemical and Biological Engineering, Hanbat National University, 125 Dongseo-daero, Yuseong-gu, Daejeon 34158, Republic of Korea

[†] These authors contributed equally to this work.

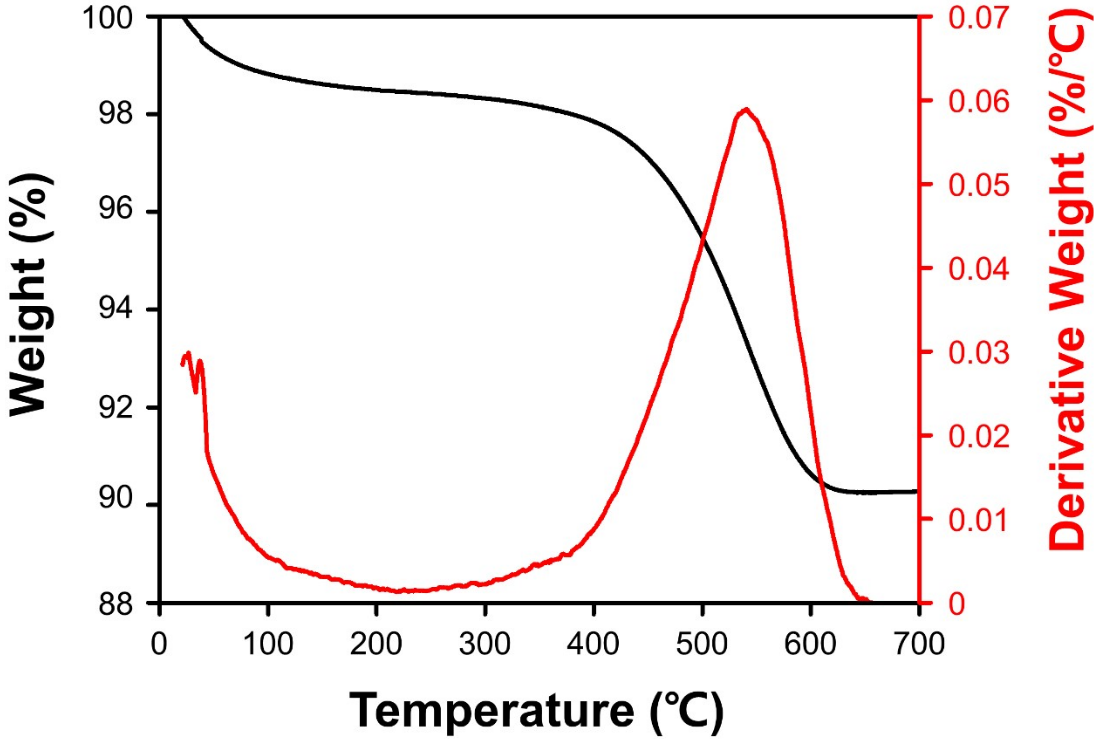

Figure S1. Thermogravimetric analysis (TGA) data of the fully deactivated $MFI_{TPAOH/(TEOS+Fe)}$ catalyst after the methanol-to-hydrocarbon reaction. The data was obtained by flowing the 40:60 mixture of N_2 and air gases by 100 cm^3 min^{-1}, while heating the sample to 973 K at a ramping rate of 10 K min^{-1}.

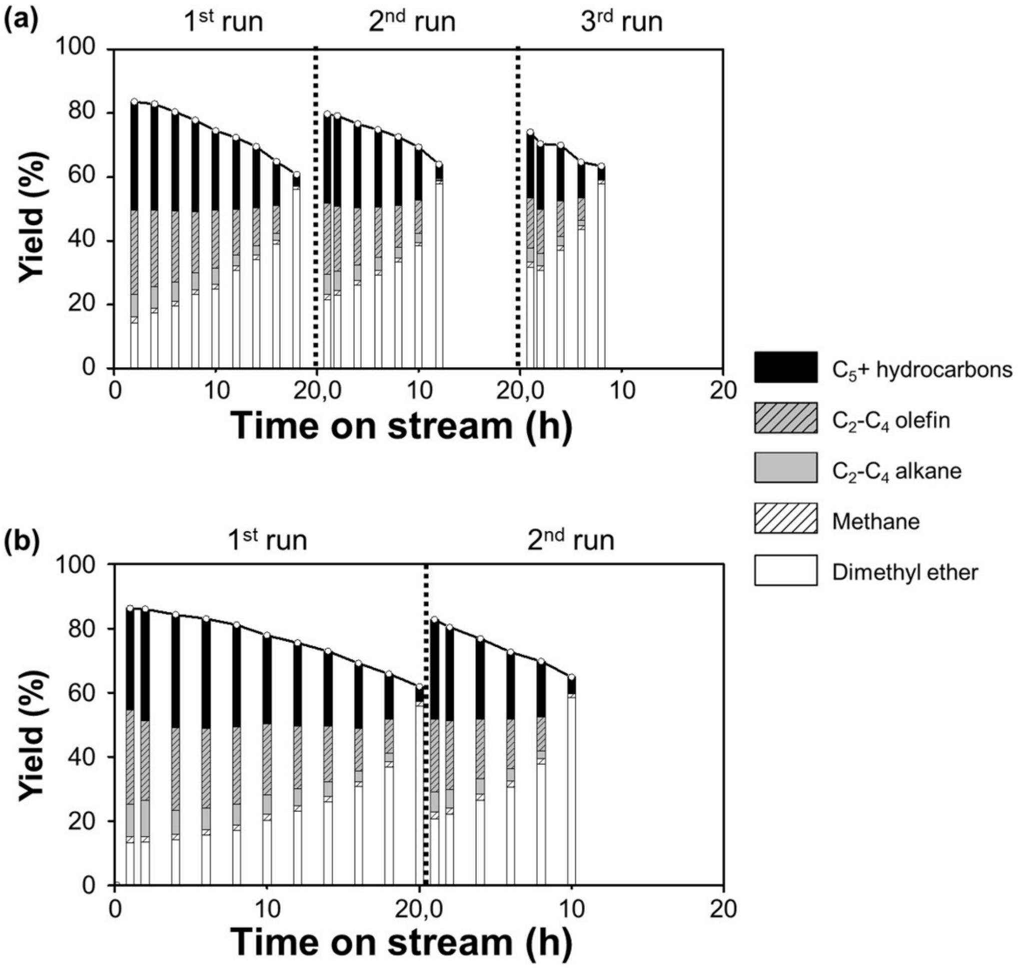

Figure S2. Regenerations of the $MFI_{TPAOH/(TEOS+Fe)}$ catalyst in the methanol-to-hydrocarbon reaction. Reaction conditions were as follows: 50 mg catalyst, 400 °C, and WHSV of methanol = 50 h^{-1}. The reactivation conditions were as follows: (a) flowing 2 vol% O_2 in N_2 under 500 °C for 2 h, (b) flowing air under 550 °C for 1 h.

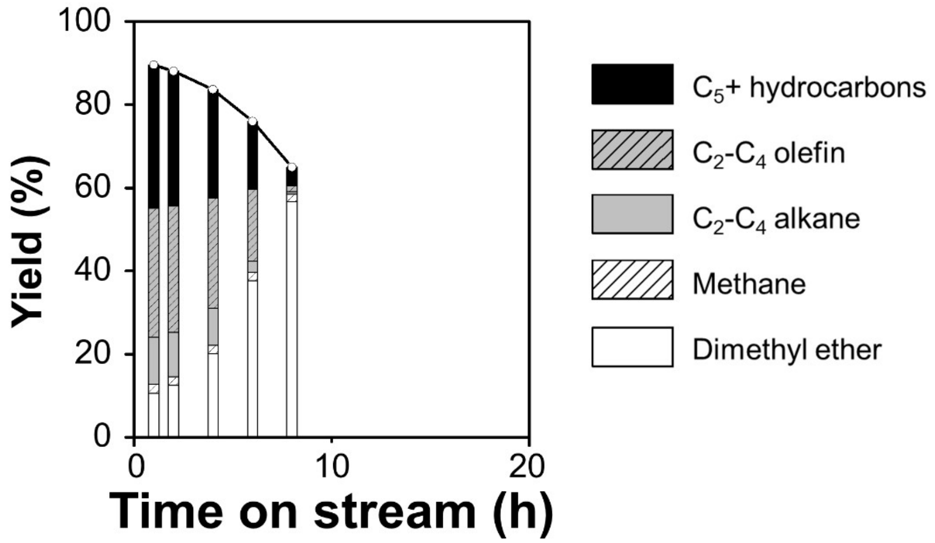

Figure S3. The MTH reaction catalytic performance of Fe-MFI synthesized by dropwisely adding H_2O-TEOS-TPAOH mixture into iron nitrate solution.[36] Reaction conditions were as follows: 50 mg catalyst, 400 ℃, and WHSV of methanol = 10 h^{-1}.

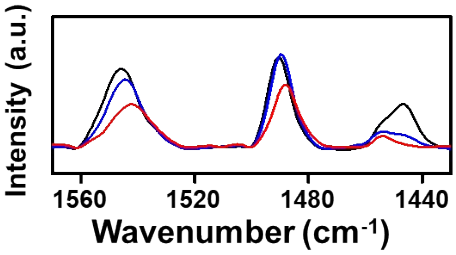

Zeolite sample	Brønsted acid site (μmol/g of zeolite)		
	150 °C	300 °C	500 °C
Al-MFI (Si/Al=40)	174	148	102

Figure S4. FT-IR spectra of pyridine-adsorbed ZSM-5, and the calculated amount of Brønsted acid sites after the desorption at each temperature. Spectra were measured after the desorption of physisorbed pyridine at 150 °C (black line), 300 °C (blue line) and 500 °C (red line) for 1 h.

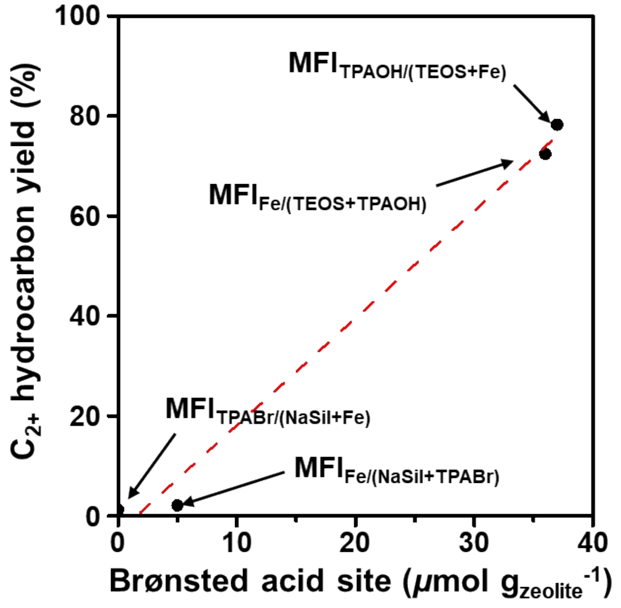

Fig. S5. The amount of Brønsted acid sites of Fe-MFI zeolites measured after the desorption of pyridine at 500 °C, and their C_{2+} hydrocarbons yield at 4 h of reaction time-on-stream.

Table S1. Relative crystallinity in comparison to the case of purely siliceous MFI zeolite.

	$MFI_{TPAOH/(TEOS+Fe)}$	$MFI_{Fe/(TEOS+TPAOH)}$	$MFI_{Fe/(NaSil+TPABr)}$	$MFI_{TPABr/(NaSil+Fe)}$
Relative Crystallinity (%)	88.7	87.1	91.0	84.5

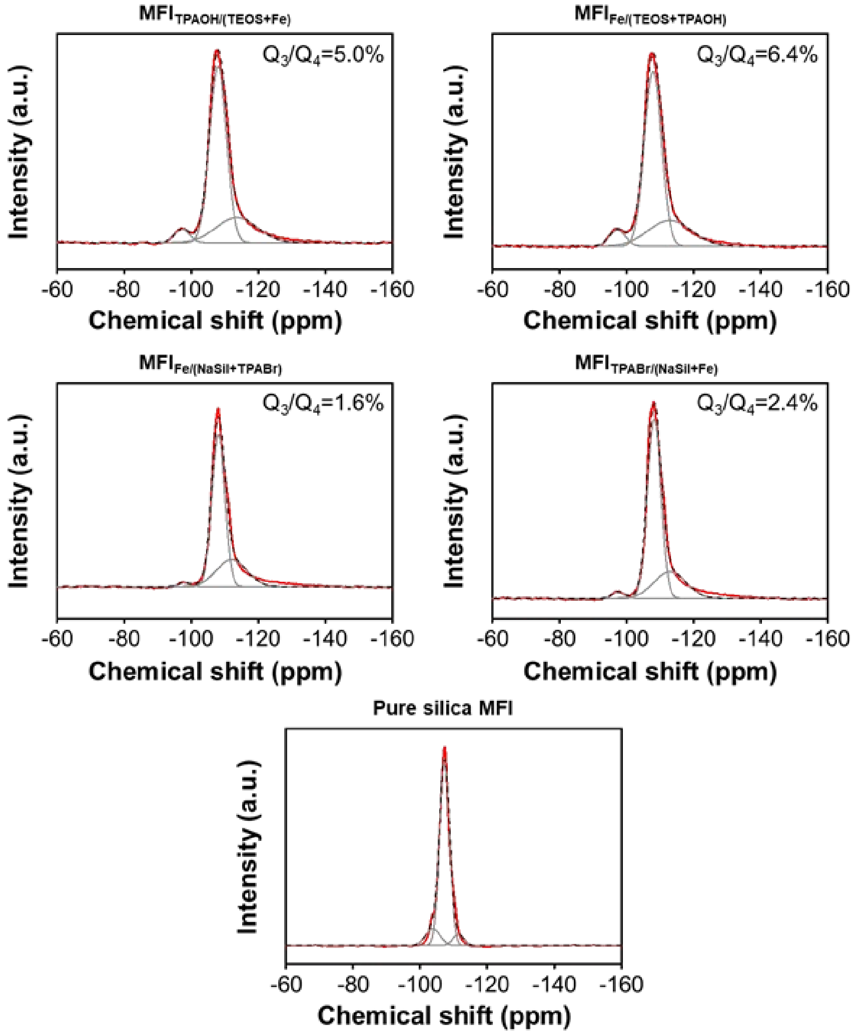

Figure S6. Solid state ^{29}Si MAS NMR spectra of the Fe-MFI zeolites, and purely siliceous MFI zeolite as reference. The measured spectra were drawn in solid red lines, and the calculated proportions of Q_3 over Q_4 were written over each NMR result. Grey curves indicate peak deconvolution, and the black dashed lines illustrate the integration of deconvoluted peaks.

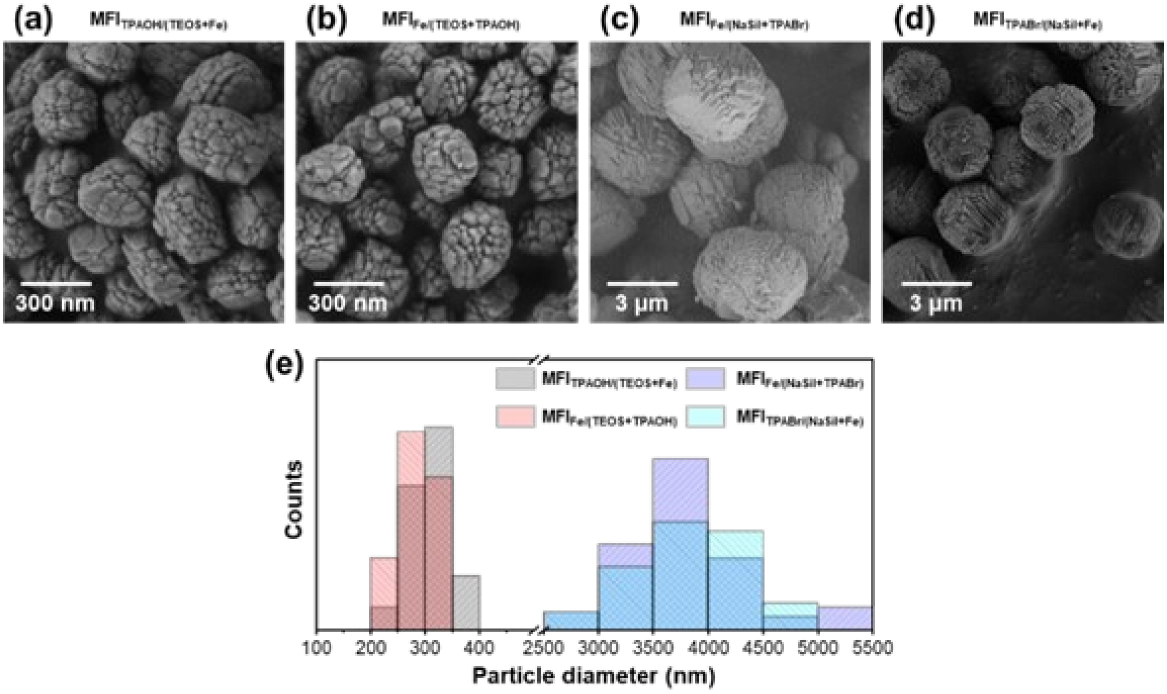

Figure S7. SEM images of (a) $MFI_{TPAOH/(TEOS+Fe)}$, (b) $MFI_{Fe/(TEOS+TPAOH)}$, (c) $MFI_{Fe/(NaSil+TPABr)}$ and (d) $MFI_{TPABr/(NaSil+Fe)}$, and the (e) particle size distributions of each sample.

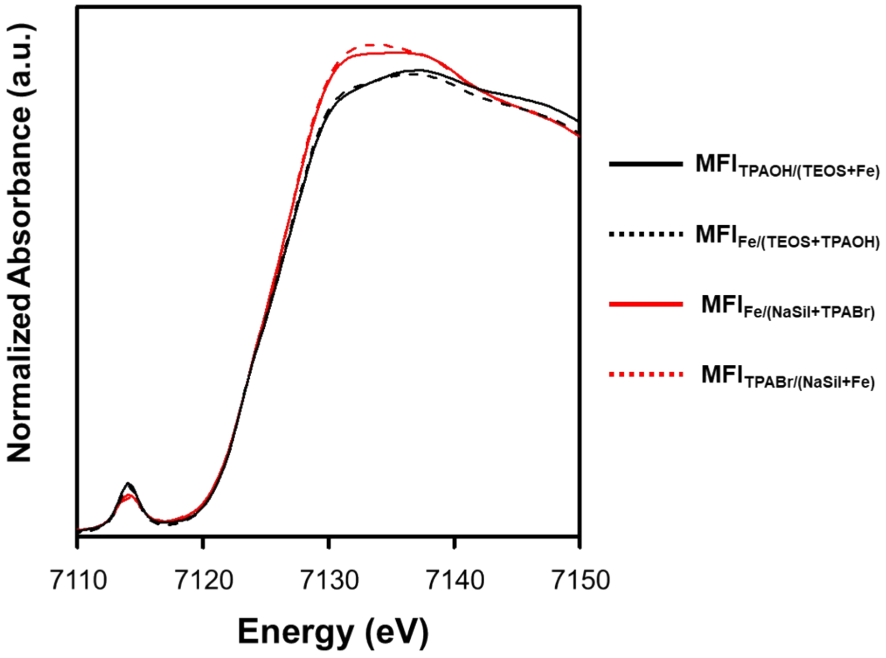

Figure S8. X-ray absorption of Fe-MFI at Fe K-edge region.
